# 

**Published:** 1921-05-21

**Authors:** 


May 21, 1921] [Price Twopence

### The Hospital

The Workers' Newspaper of Administrative Medicine and Institutional Life, Administration, National Insurance and Health.

CONTENTS

The Next Step at the "London" ... ... ... 123

School Clinics ... ... ... ... ... ... ... 123

The Crisis Once More ... ... ... ... ... 124

Is a Hospital Pageant Practicable? ... ... 124

Notes of the Week ... ... ... ... ... ... 125

Collective Propaganda—The Royal Family and the Hospitals—Dispensary Tuberculosis Treatment and the Rates—Pictures Help Hospital Appeal—New Secretary of the Guest Hospital—Better News from Bolingbroke Hospital—An Interesting Dinner—New Tuberculosis Hospital, Monmouthshire—A Deficit Cleared Off—Arklow Cottage Hospital Opened—The Sheffield Contribution Scheme—The Irish Appeal.

The Modern Treatment of Ringworm ... ... 127

The Recurrence of Rabies ... ... ... ... 127

A Plea for More Drastic Control.

"Carriers" of Disease ... ... ... ... ... 128

Parliament and the Professions ... ... ... 128

Hospital Engineering Reforms ... ... ... 129

II.—Possible Economies with Steam Boilers.

Medical Aid for Domestic Servants ... ... 130

The Employer's Liability.

The Public Well-Being ... ... ... ... ... 131

Athletics for Girls—The Elixir of Youth—Human Ideals in Industry—Government Food Pamphlets.

The Editor's Letter-Box ... ... ... ... 133

The Practice of Birth-Control—Hospital Claims and the Insurance Surplus—The Fly Pest.

Passing Events and Topics ... ... ... ... 134

The Matrons' and Sisters' Department ... ... 135

First-year Training-schools.
King Edward's Memorial Homes for Nurses.

Round the Hospitals ... ... ... ... ... 136

Annual Meetings of Hospitals ... ... ... 139

The College of Nursing (Limited by Guarantee) 139

Matrons' and Sisters' Appointments ... ... 140

